# Effectiveness of Polymer Additives in Concrete for 3D Concrete Printing Using Fly Ash

**DOI:** 10.3390/polym14245467

**Published:** 2022-12-13

**Authors:** Leonid Dvorkin, Janusz Konkol, Vitaliy Marchuk, Andriy Huts

**Affiliations:** 1 Institute of Civil Engineering and Architecture, National University of Water and Environmental Engineering, 33028 Rivne, Ukraine; 2 Faculty of Civil and Environmental Engineering and Architecture, Rzeszow University of Technology, 35959 Rzeszow, Poland

**Keywords:** Portland cement, concrete, redispersible polymer powder, 3D construction printer, mathematical experiment planning, hardening accelerator

## Abstract

The article shows the effectiveness of the use of polymer additives for the production of fine-grained concrete mixtures and concretes based on using coal fly ash, which can be used as working mixtures for a 3D printer. Using mathematical planning of experiments, a set of experimental–statistical models was obtained that describes the influence of mixture composition factors including copolymer additive on the most important properties of ash-containing concrete mixtures and concretes for 3D concrete printing in the presence of a hardening accelerator additive. It is shown that when the dry mixture is mixed in water, the redispersed polymer powders are converted into an adhesive polymer dispersion, which, when the solution cures, creates “rubber bridges” in its pores and at the border with the base. They have high tensile strength and elastically reinforce the cement stone; in addition, they are also capable of not only significantly increasing the adhesion between the layers of the extruded mixture, but also significantly smoothing out such shortcomings of the cement stone as increased brittleness, low ultimate elongation, and a tendency to cracking.

## 1. Introduction

Modern construction is developing at a high speed due to the integration of innovative technologies and modern building materials into its process. The construction industry is one of the most resource- and energy-consuming, since the construction of buildings and structures for industrial and civil purposes requires a significant amount of non-renewable resources, the consumption of which increases with the increasing needs of people [1]. Today, concrete remains the main structural material in the construction sector; thus, the priority is to reduce the energy intensity of its production, which corresponds to the global concept of low-carbon development (sustainable development) and the reduction of CO_2_ levels, the vector of which is the rational use and saving of material and energy resources. The development of such materials makes it possible to rationally use natural raw materials, fuel, electrical energy, utilize production waste, and reduce greenhouse gas emissions. This approach allows solving a number of important environmental, economic, and social problems [2]. From the standpoint of environmental safety, additive technologies can also be effective, allowing to realize high production efficiency, improving the safety of work at the construction site [3,4].

The most famous additive technology is the use of 3D printing in the construction process. The essence of this method is the layer-by-layer application of the mixture. With the use of 3D printers, it is possible to provide high-speed robotic construction of objects, including complex shapes, with a minimization of material consumption and labor use.

Nowadays, a large amount of materials for 3D printing is used; the main ones are fine-grained concrete mixtures using Portland cement, mineral aggregates and fillers, various chemical additives, and fiber [5,6].

Building mixtures for 3D printing must have certain properties, in particular: the necessary workability (extrudability); structural strength after a certain time of layer curing; tensile strength at splitting, characterized by adhesion between the layers; as well as design compressive strength.

A series of studies indicate the improvement of the properties of mixtures suitable for 3D concrete printing by adding various polymer additives to the cement matrix [7,8]. Their effect on the structure leads to the improvement of the properties of concrete mixtures and hardened concrete, such as workability [9], setting time [10], frost resistance [11], and water impermeability [12]. The use of polymers to increase the adhesion strength of concrete is associated with changes in the microstructure and the effect of reducing shrinkage [13,14,15].

During mixing in water, redispersed polymer powders are transformed into an adhesive polymer dispersion, which, when the concrete cures, creates “rubber bridges” in its pores and at the interface with the base [7]. They have high tensile strength and elastically reinforce cement stone [16]; in addition, they can not only significantly increase the adhesion of concrete to the base, but also significantly reduce the disadvantages of cement stone, such as increased fragility and tendency to cracking [17]. It is necessary to note that the polymer does not chemically interact with binders and other components; however, it only plays the role of flexible bonds, giving the cement stone increased elasticity. With the use of dispersion powders, some special properties are also provided to used mixtures [12,13,14].

Yi Zhang et al. [8] investigated the effect of redispersed polymer powder (RPP) on the formability and structural strength of cement mixtures suitable for 3D printing. Their research showed that RPPs have a positive effect on the properties of extruded cement mixture during dynamic and static tests. In addition, it is known that the polymer can increase the durability of such concretes, which is an important aspect to consider in construction [18,19].

Nowadays, the technology of cement concrete and mortars using industrial wastes, in particular fly ash, has been developed. Fly ash, having a high specific surface area, in addition to a direct chemical interaction with cement, actively affects the physicochemical processes on the surface of distribution “cement paste—aggregate” where the formation of contacts between them begins. For ash-containing mixtures, the impact of RPP is currently not fully investigated.

The aim of this work was to develop ash-containing mixtures using redispersed polymer powder, characterized by the required technological properties, as well as structural strength at a certain time after extrusion of the layer, strength, providing adhesion of concrete layers, and compressive strength at an early and a project age.

## 2. Materials and Methods

The materials used in the research were Portland cement CEM I 42.5R of Cement Plant Dyckerhoff Ukraine and fly ash from Burshtynska thermal power plant (Burshtyn, Ukraine), which belongs to type II ash of category B with a residue on a sieve, with a mesh size of 45 μm no more than 25% (class 2) (EN 450-1:2012). The chemical composition of Portland cement and fly ash are given in Table 1. The mineralogical composition of the clinker was as follows: C_3_S—57.10%; C_2_S—21.27%; C_3_A—6.87%; C_4_AF—12.19% (EN 196-2). The specific surface area of the Portland cement was S = 300–320 m^2^/kg (EN 196-6).

Quartz sand with fineness modulus 2.1 was used as an aggregate. Chemical additives were: superplasticizer (SP) of polycarboxylate type and hardening accelerator (HA) sodium sulfate Na_2_SO_4_. The redispersed polymer powders are selected among the most popular ones, in particular: vinyl ester of versatile acid (VEOVA), copolymer—vinyl acetate–ethylene (VAE), and polymer of vinyl acetate (PVA), the characteristics of which are given in Table 2.

**Figure 1:**
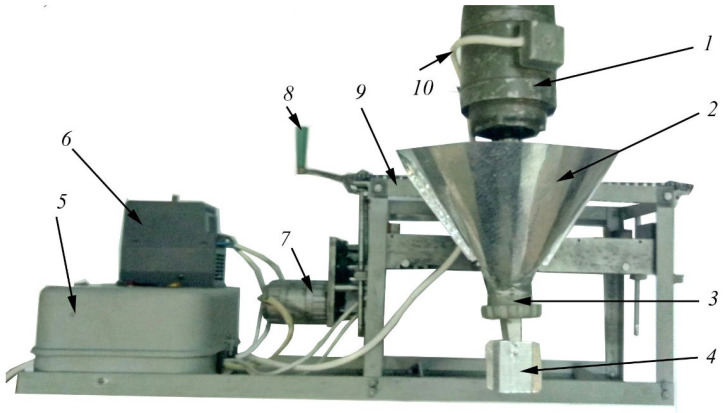
Laboratory 3D printer. 1—electric motor of the extruder; 2—hopper of building mixture; 3—auger; 4—mouthpiece; 5—control panel; 6—frequency converter of electricity; 7—reverse motor moving the extruder in the horizontal direction: 8—manual drive moving the extruder in the vertical direction; 9—frame; 10—power cable of electric motors.

The main task in the study of suitability for 3D printer fine-grained concrete mixtures without coarse aggregate DSTU B V.2.7-43-96 (Ukrainian Standart) is to ensure their required shape when extruded from the printer head with the achievement of the specified structural strength, as well as the strength of adhesion between the layers without the formation of cracks and other defects [5,20,21]. Samples for determining the properties of mixtures were made using a laboratory 3D printer (Figure 1), the characteristics of which are given in [5].

The 3D printed sample was cut into four-layer specimens with cross-sectional dimensions of 40 mm × 50 mm × 50 mm. The surfaces of the samples to which the load was applied were additionally smoothed.

The workability of concrete mixtures was determined by immersion of a standard cone; the tensile splitting strength and compressive strength were determined according to EN 196-1 at the age of 1, 7, and 28 days. The testing machine FP-100/1 100 kN (VEB Fritz–Heckert–Werk, Chemnitz, Germany) was used for testing. Tests for tensile splitting strength were carried out on three samples. The samples were placed in the machine and loaded with cylindrical steel heads at the boundaries of the layers. The load was transferred to the sample at a constant speed of 50 N/s until destruction. Compressive strength was tested on six samples; the load was transferred to the sample at a speed of 500 N/s until destruction. The head of the testing machine was assembled with a hinge to adapt to any possible non-parallel surface.

The setting time (curing time) was determined according to EN 196–3 by the time from the moment of mixing to the beginning of setting, at which it becomes impossible to further mold with a 3D printer.

**Figure 2:**
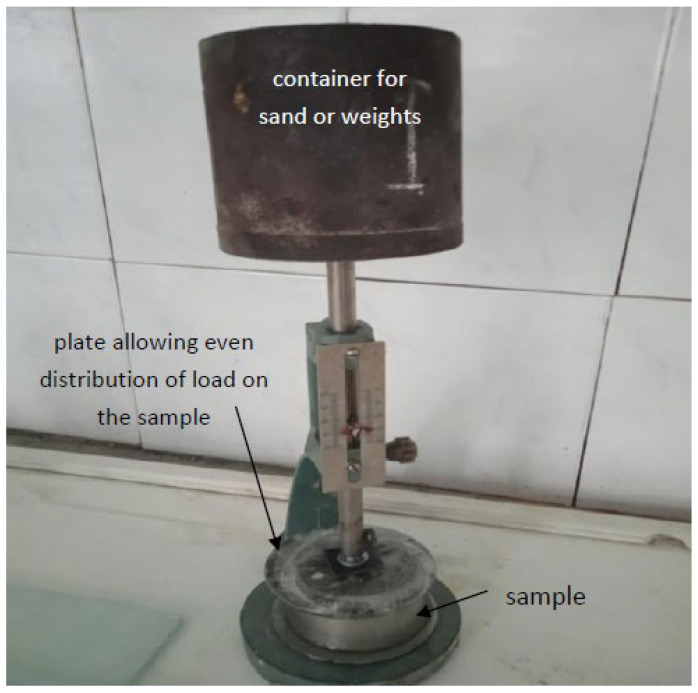
Device for determining the structural strength.

**Figure 3:**
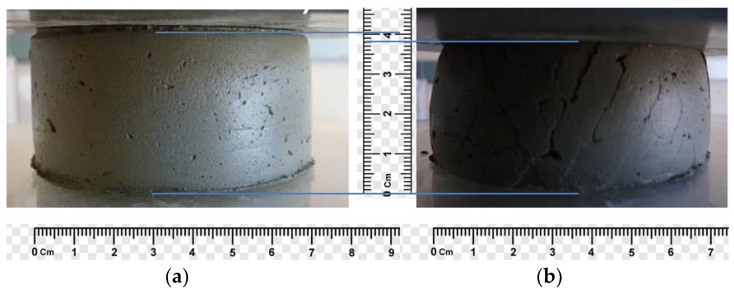
An example of determining the structural strength of extruded concrete: (**a**) the sample withstands the load (structural strength is provided); and (**b**) the sample is destroyed.

To determine the structural strength, a technique is proposed that allows to measure the ultimate load limit on a sample of an extruded concrete layer, at which it begins to deform [5] (Figure 2 and Figure 3).

## 3. Results and Discussion

At the first stage of the study, the effects of redispersed polymer powders of different natures were compared. In the mixtures containing cement and quartz sand, a polycarboxylate superplasticizer and a hardening accelerator were added during mixing. The proportion of polymer was 0%, 1.0%, and 2.0% by weight of the dry mix; the W/C ratio was constant at each point (W/C = 0.6). The compositions of the studied mixtures are given in Table 3.

**Figure 4:**
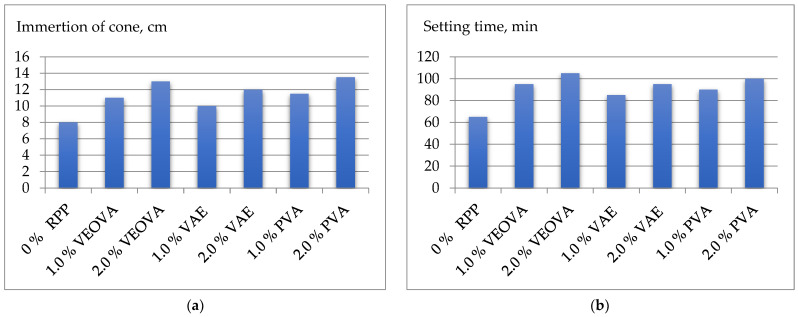
Properties of mixtures using RPP: (**a**) workability; (**b**) setting time.

**Figure 5:**
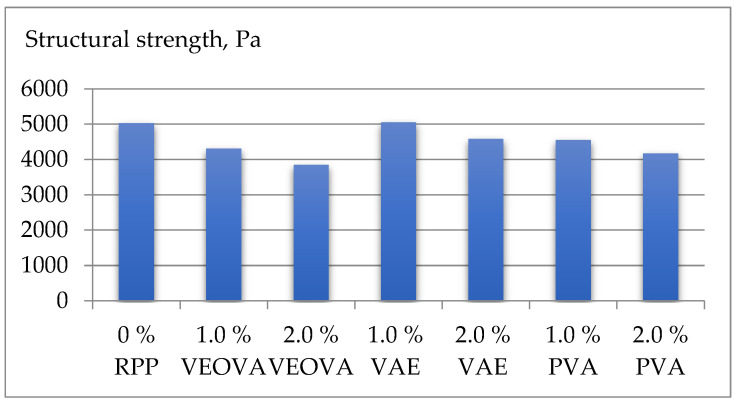
Structural strength in 30 min after mixing mixtures using RPP.

**Figure 6:**
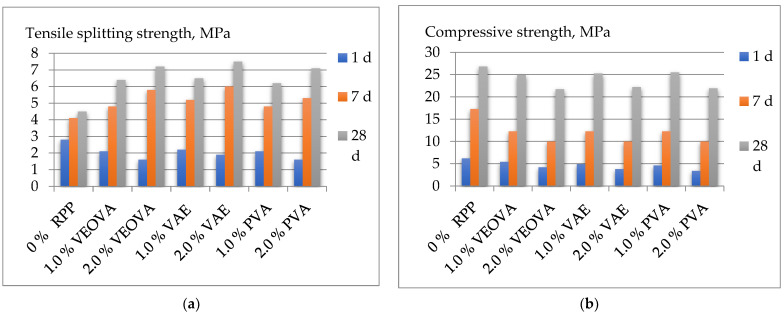
Results of concrete strength with the addition of RPP: (**a**) tensile splitting strength; (**b**) compressive strength.

The obtained experimental results are shown in Table 4 and Figure 4, Figure 5 and Figure 6.

Analyzing the results obtained, it can be concluded that the addition of RPP causes an increase in the workability of the mixture on the immersion of the cone from 8 cm to 10–11.5 cm at a constant water content and polymer content of 1% by weight of the mixture. With a further increase in the amount of RPP to 2%, there is a slight increase in workability to 12–13.5 cm (Figure 4a). The effect on the setting time is similar (Figure 4b).

The effect of RPP on the structural strength 30 min after mixing the mixture is ambiguous. Vinyl ester of versatic acid significantly reduces structural strength. The effect of polyvinyl acetate is similar; however, the reduction of strength is less significant. At the same time, a positive effect is observed at a medium content (1%) of the vinyl acetate–ethylene copolymer; however, with a further increase in the content of RPP, the structural strength also decreases compared to the sample without polymer powder (Figure 5).

Polymer powders have mixed effects on both compressive and splitting strengths at different curing times. According to the results obtained (Table 4 and Figure 5), the addition of polymers of all types significantly reduces the tensile splitting strength and compressive strength at the early stages of curing. However, when approaching 28 days of age, RPPs have a positive effect on the studied parameters.

The tensile splitting strength (Figure 6a) at the age of 7 days increases in comparison with the mixture without a polymer additive by 15–25% and 30–40% with the addition of RPP of 1.0% and 2.0%, respectively. At the age of 28 days, the increase is more significant from 35–40% to 55–65% with a respective increase in the additive from 1.0% to 2.0%. The highest results (7.5 MPa) were obtained using a copolymer—vinyl acetate–ethylene in the amount of 2.0% by weight of the dry mixture.

The effect of RPP on compressive strength (Figure 6b) is somewhat different. It has a mostly negative effect, with an addition of up to 1.0%; at the age of 28 days, the strength decreases by 7–10%. With a further increase of polymer content of up to 2.0% in the mixture, the strength decreases by 16–20%. The effect on early strength, in comparison with the 28-day strength, is more significant and is accompanied with a decrease in strength compared to the sample without a polymer additive. The impact of polymers on the mechanical properties of cement systems is mainly due to their adsorption effect, which depends on the characteristics of the polymer structure. RPP reduces the rate of hydration at an early age [22,23,24,25].

Thus, it can be concluded that the best results of structural strength and tensile splitting strength, which characterizes the adhesion between the layers, were obtained using a copolymer–vinyl acetate–ethylene; thus, further studies were carried out using this RPP.

In the second stage, algorithmic experiments were performed to study the combined effect of fly ash and vinyl acetate–ethylene copolymer on the properties of concrete suitable for 3D printer according to the three-level two-factor plan B_2_ [26]. The use of mathematical planning of experiments allowed to algorithmize the experiments according to the scheme, which is optimal in terms of both the volume of experimental work and statistical requirements. The experiment was planned in accordance with a typical matrix, i.e., a table with *n* rows and *m* columns, which gives a set of combinations of factors varied relative to some origin or zero (basic) level. The permissible range of the variation of factors (factor space) is selected on the basis of a preliminary study of the object in accordance with the purpose. To simplify the recording of experimental conditions and processing of experimental data, the upper level of factors is coded +1, the lower level −1, and the main level corresponds to 0.

For the construction of quadratic models, a full factorial experiment was used, which provides for all the possible combinations of factors at three levels. For the technological analysis and selection of significant factors, along with checking the adequacy of the equation, the significance of the regression coefficients was also assessed. The significance of the regression coefficients *b_i_* was estimated by finding the experimental value of the *t*—criterion (*t_i_*) and comparing it with the table [26]. Regression equations, having a quadratic character, allow to trace the individual and combined influence of factors on the studied output parameters, to establish the necessary and optimal values of factors.

The results of the experiments were processed using methods of mathematical statistics, obtaining quadratic regression equations in general form for *k* factors. The conditions for planning the experiments are given in Table 5.

The parameters studied were: the setting time; structural strength 30 min after mixing; compressive strength; and tensile splitting strength at the age of 1, 7, and 28 days.

The planning matrix and composition of the mixtures, as well as experimental results are given in Table 6, Table 7 and Table 8.

During the research, at each point of the plan to evaluate the influence of factors on the properties of concrete, mixtures were prepared according to Table 6. The fly ash content in the cement–ash binder (CAB) was constant and amounted to 30% by weight of the binder. Additionally, a polycarboxylate superplasticizer in the amount of 0.3% and a hardening accelerator of 1% by weight of cement were added.

The water consumption was varied to ensure the necessary workability by immersion of a standard cone of 8–10 cm, which ensured sufficient formability (extrusion) of the mixture. Formability was determined by the ability of the mixture to be squeezed out of the printer’s mouthpiece without cracks and delaminating along the length of the bar.

The suitability of the mixtures for molding was determined by the time from the moment of mixing to the beginning of hardening after which further molding on a 3D printer becomes impossible.

After statistical processing of the results of the experiments (Table 7), performed according to [26], the coefficients of the regression equations of the setting time; structural strength; as well as compressive strength, and tensile splitting strength of the studied mixtures and hardened concrete were obtained (Table 8), which can be considered as characteristics of the influence of the studied factors on the quality indicators of concrete properties in a certain range of their variation.

General type of equations:

(1) $$Y=\beta_{0}+\overset{n}{\underset{i=1}{\Sigma }}\beta_{i}X_{i}+\overset{n}{\underset{i=1}{\Sigma }}\beta_{ii}X_{i}^{2}+\sum_{i\neq j}\beta_{ij}X_{i}X_{j}+\ldots$$

where *Y* is the calculated value of the studied parameter; *X_i_*…*X_j_* are independent variables (factors) that can be varied during experiments; *β_0_*, *β_i_*…*β_j_*, *β_ii_*…*β_ij_* are statistical estimates of regression coefficients.

The analysis of the coefficients (Table 8) of the regression equations (Equation (1)) of the indicators of the properties of concrete suitable for the 3D printer allows to evaluate the factors by the magnitude of the effect of their influence. The studied properties are more significantly affected by the content of CAB compared to the addition of redispersed polymer powder with an increase in their consumption in the range of variation. According to the results, there is a significant influence on the interaction effects of factors. Obviously, achieving high physical and mechanical properties of concrete is possible with appropriate optimization of the content of CAB and RPP in their mixtures.

**Figure 7:**
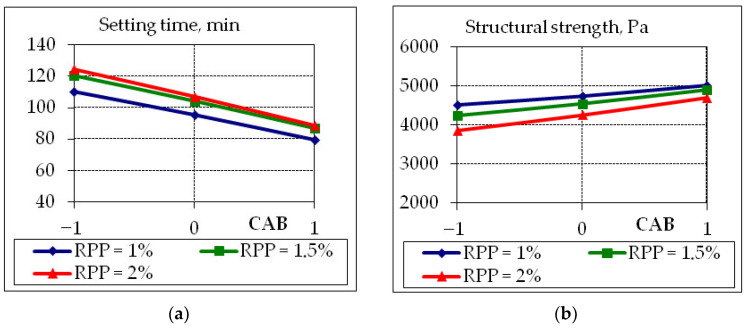
Graphical dependences of setting time (**a**) and structural strength (**b**) 30 min after mixing of fly ash concrete suitable for 3D printer, where CAB content −1 = 15%; 0 = 17.5%; +1 = 20%.

**Figure 8:**
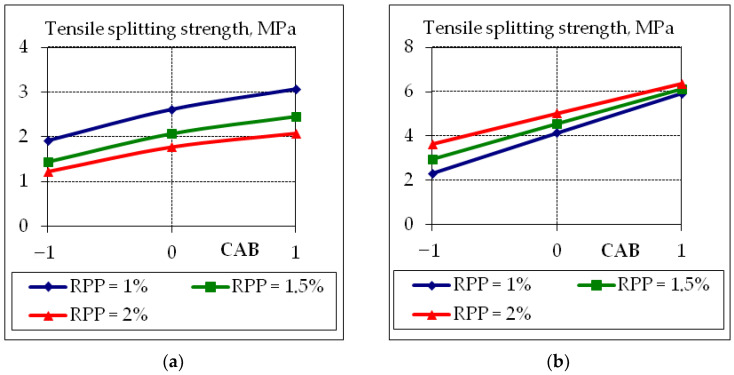
Graphical dependences of tensile splitting strength at the age of 1 day (**a**) and 28 days (**b**) of fly ash concrete suitable for 3D printer, where the content of CAB −1 = 15%; 0 = 17.5%; +1 = 20%.

**Figure 9:**
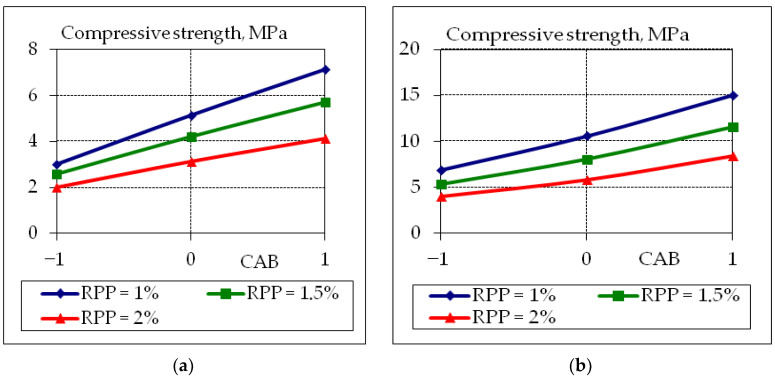
Graphical dependences of compressive strength at the age of 1 day (**a**) and 7 days (**b**) of fly ash concrete suitable for 3D printer, where the content of CAB −1 = 15%; 0 = 17.5%; +1 = 20%.

**Figure 10:**
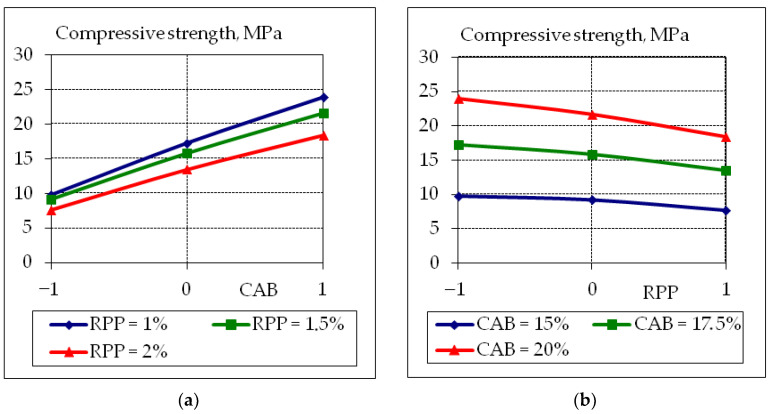
Graphical dependences of compressive strength at the age of 28 days of fly ash concrete suitable for 3D printer, where the content of CAB (**a**) −1 = 15%; 0 = 17.5%; +1 = 20%, and RPP (**b**) −1 = 1.0%; 0 = 1.5%; +1 = 2.0%.

To analyze the obtained experimental and statistical models, two-factor graphical dependencies were built, which show the influence of composition factors on the properties of concrete suitable for the 3D printer (Figure 7, Figure 8, Figure 9 and Figure 10).

The addition of RPP to the mixtures for the 3D printer allows to increase the setting time (Figure 7a) or the so-called “printing window” by 8–15%. However, this has a negative consequence associated with a decrease in structural strength, which decreases at the maximum water consumption within the experiments (Figure 7b). Structural strength increases more significantly by 12–15% with an increase in CAB content compared to its decrease with an increase in the amount of RPP by 8–10%.

The obtained results clearly show the negative effect on the tensile splitting strength (Figure 8a) and compressive strength (Figure 9a) of concrete at the age of 1 day with the increasing of the content of the RPP additive (down to 50%). However, it should be noted that with increasing polymer content, there is a positive effect on the tensile splitting strength at the age of 28 days (Figure 8b) increase by 8–12% (from 2.2 MPa to 3.8 MPa) and 35–40% (from 5.95 MPa to 6.5 MPa) at CAB consumption 20% and 15%, respectively. Polymers reduce the rate of hydration at an early age; thus, reducing the amount of calcium hydroxide that binds to fly ash, forming additional neoplasms that affect the properties of mixtures suitable for 3D printers; these statements are confirmed in [22,23,24,25].

The influence of the studied factors on the strength parameters is to some extent linear, as evidenced by the insignificant quadratic coefficients of the regression equations (Table 8). This is especially characteristic for the tensile splitting strength at the age of 28 days and compressive strength at all curing times (Figure 9 and Figure 10). The increase in tensile splitting strength due to the increase in CAB consumption is less significant at 1% RPP content consumption strength increase by 35–40% at 20% CAB consumption and 8–12% at 15% CAB.

## 4. Conclusions

The purpose of fly ash in construction mixtures and RPP is different; however, their combination gives a positive effect. Fly ash is an active mineral additive that helps to increase the volume of hydrate formations; moreover, the addition of fly ash to cements and concrete leads to a reduction in clinker consumption, which reduces CO_2_ emissions and improves the environmental situation. RPP, in turn, increases the plasticity (extrudability) of the mixture, as well as the adhesive strength of the adhesion of the layers, which is important for mixtures used in 3D printers.

Comparing the analysis of the influence of the studied redispersible polymer powders allows to consider that the best values of the complex of basic properties of mixtures and concretes for a 3D printer are achieved when using copolymer–vinyl acetate–ethylene.

Addition of redispersible polymer powder to the fly ash containing concrete mixture increases the tensile splitting strength, which improves the adhesion strength between the layers of the printed mixture.

As follows from the nature of the interaction between redispersible polymer powder and cement–ash binder, the increased content of polymer additive has a more positive effect on the tensile splitting strength at a lower cement–ash binder consumption strength increase by 35–40% at 20% CAB consumption and 8–12% at 15% CAB.

The use of a redispersible polymer powder with a cement–ash binder content of 180–200 kg/t, along with increased adhesive strength, makes it possible to provide the necessary compressive strength of ash-containing concrete, suitable for printing on a 3D printer.

In further research, it is advisable to develop compositions of building mixtures for a 3D printer using other dispersed products of technogenic origin; in addition, to offer technological solutions that take into account the design features of existing 3D printers.

## Figures and Tables

**Table 1:** Chemical composition of Portland cement *.

Name of Material	L.O.I.	Oxide Content, %							
		SiO_2_	Al_2_O_3_	Fe_2_O_3_	CaO	MgO	SO_3_	K_2_O	Na_2_O
**Clinker**	-	21.80	5.32	4.11	66.80	0.95	0.63	0.54	0.42
Fly ash	5.1	46.1	18.1	22.1	2.1	2.0	2.3	1.2	

* The chemical composition of Portland cement based on the used clinker was distinguished by an additional SO_3_ content due to the introduction of gypsum in the amount of 3.1%.

**Table 2:** Polymers characteristics.

Polymer	Ash Content, %	Minimum Temperature of Film Formation, °C	pH
VEOVA	10–14	6	5–6
VAE	8–12	−10	7
PVA	6–10	5	5–6

**Table 3:** Compositions of the studied mixtures suitable for 3D printer using RPP.

No.	Polymer	Dry Mix Composition, kg/t					Mixing Water, L/t of Mixture
		RPP	Cement	Sand	SP	HA	
1	–	0	200	797.4	0.6	2	120
2	VEOVA	10	200	787.4	0.6	2	120
3	VEOVA	20	200	777.4	0.6	2	120
4	VAE	10	200	787.4	0.6	2	120
5	VAE	20	200	777.4	0.6	2	120
6	PVA	10	200	787.4	0.6	2	120
7	PVA	20	200	777.4	0.6	2	120

**Table 4:** Results of influence of RPP on the properties of concrete.

No.	Immersion of the Cone, cm	Setting Time, min	Structural Strength after 30 Min after Mixing, Pa	Tensile Splitting Strength at Age, MPa			Compressive Strength at Age, MPa		
				1 Day	7 Days	28 Days	1 Day	7 Days	28 Days
1	8.0	65	5030	2.8	4.1	4.5	6.2	17.3	26.8
2	11.0	95	4310	2.1	4.8	6.4	5.4	12.3	24.9
3	13.0	105	3850	1.6	5.8	7.2	4.2	10.0	21.7
4	10.0	85	5050	2.2	5.4	6.5	4.9	12.3	25.2
5	12.0	95	4580	1.9	6.1	7.5	3.8	10.0	22.2
6	11.5	90	4550	2.1	4.8	6.2	4.6	12.3	25.5
7	13.5	100	4170	1.6	5.1	7.1	3.4	10.0	21.9

**Table 5:** Conditions for planning experiments of the study.

Technological Factors		Levels of Variation			Variation Interval
Natural View	Coded View	−1	0	+1	
Proportion of cement–ash binder (CAB), % by weight	X_1_	15	17.5	20	2.5
Redispersible polymer powder content (RPP), % by weight	X_2_	1.0	1.5	2.0	0.5

**Table 6:** Planning matrix and composition of mixtures.

No.	Coded View		Natural View		Material Content per 1 t of Dry Mix				Mixing Water, L
	X_1_	X_2_	CAB, %	RPP, %	CEM, kg	Fly Ash, kg	RPP, kg	Sand, kg	
1	1	1	20	2.0	140	60	20	780	130
2	1	−1	20	1.0	140	60	10	790	136
3	−1	1	15	2.0	105	45	20	830	118
4	−1	−1	15	1.0	105	45	10	840	130
5	1	0	20	1.5	140	60	15	785	133
6	−1	0	15	1.5	105	45	15	835	125
7	0	1	17.5	2.0	123	53	20	805	126
8	0	−1	17.5	1.0	123	53	10	815	133
9	0	0	17.5	1.5	123	53	15	810	131
10	0	0	17.5	1.5	123	53	15	810	131
11	0	0	17.5	1.5	123	53	15	810	131

**Table 7:** Experimental results of research.

No.	Setting Time, min	Structural Strength, Pa	Strength, MPa					
			Tensile Splitting, at Age			Compressive, at Age		
			1 Day	7 Days	28 Days	1 Day	7 Days	28 Days
1	90	4710	2.1	4.3	6.5	4.0	8.0	18.0
2	80	5020	3.1	3.4	6.0	7.0	15.0	24.0
3	125	3880	1.2	2.7	3.7	2.0	4.2	7.5
4	110	4520	1.9	2.1	2.2	3.0	7.5	10.0
5	85	4920	2.4	3.6	6.0	6.0	12.0	22.0
6	120	4220	1.5	1.9	3.0	2.6	4.5	9.1
7	105	4240	1.8	3.9	4.9	3.3	6.0	14.0
8	95	4750	2.6	2.9	4.2	5.3	10.0	17.0
9	105	4550	2.1	3.2	4.6	4.3	8.5	15.2
10	105	4570	2.1	3.3	4.6	4.0	8.0	16.1
11	105	4530	2.0	3.2	4.5	4.0	8.0	16.0

**Table 8:** Coefficients of regression equations.

Coefficients	Setting Time, min	Structural Strength, Pa	Tensile Splitting Strength, MPa, at Age			Compressive Strength, MPa, at Age		
			1 Day	7 Days	28 Days	1 Day	7 Days	28 Days
B_0_	104.25	4548	2.06	3.19	4.55	4.19	8.05	15.77
B_1_	−16.67	338	0.50	0.77	1.58	1.57	3.13	6.25
B_2_	5.83	−243	−0.42	0.42	0.44	−1.00	−2.38	−1.92
B_11_	−0.58	27	0.20	−0.38	−0.02	−0.06	0.39	−0.42
B_22_	−3.08	−48	0.14	0.27	0.03	−0.06	0.14	−0.42
B_12_	−1.25	82	−0.08	0.08	−0.22	−0.05	−0.93	−0.88

## Data Availability

Not applicable.
